# Use of Recycled Plastic Waste from Electrical Cable Recycling Processes as Fillers in Concrete for Paving Block Production and the Associated Slip Risk

**DOI:** 10.3390/ma19091828

**Published:** 2026-04-29

**Authors:** Marcin Giedrowicz, Bartosz Wieczorek, Konrad Jan Waluś, Miłosz Płachetka, Łukasz Warguła

**Affiliations:** 1 Institute of Architecture and Physical Planning, Faculty of Architecture, Poznan University of Technology, Jacka Rychlewskiego 1, 61-131 Poznań, Poland; marcin.giedrowicz@put.poznan.pl; 2 Institute of Machine Design, Faculty of Mechanical Engineering, Poznan University of Technology, Piotrowo 3, 61-138 Poznań, Poland; bartosz.wieczorek@put.poznan.pl (B.W.); konrad.walus@put.poznan.pl (K.J.W.); 3 MP. POLRAJ Ogrodzenia Betonowe, Długa 21, 64-514 Pamiątkowo, Poland; milosz.plachetka@gmail.com

**Keywords:** tribological performance, surface roughness, laitance effect, pedestrian safety, polymer–cement interface, Skid Resistance Value, British Pendulum Tester, recycled cable insulation, concrete paving blocks, aggregate replacement

## Abstract

**Highlights:**

- Recycled cable insulation reduces concrete mass by up to 20%;
- GCI increases slip resistance on polished concrete surfaces;
- Optimal slip performance observed at ~10% GCI for ground surfaces;
- High GCI content (>30–35%) leads to mechanical degradation;
- Slip and damage boundaries defined for safe material application.

**Abstract:**

The use of plastic waste as a filler in concrete, particularly in paving block production, represents an approach aligned with circular economy principles. While previous studies have focused on mechanical properties, the effect of such materials on slip risk remains insufficiently investigated, especially for pedestrian applications. This study evaluates the influence of the volumetric content of recycled plastic waste from electrical cable insulation on slip resistance of concrete paving blocks. A series of specimens was prepared with 0–45% replacement of natural aggregate by granulated cable insulation (GCI). Slip resistance was measured using the British Pendulum Tester and expressed as Skid Resistance Value (SRV) after statistical processing. Two sliders were used, Mounted Shoe 55 and Mounted Shoe 96, corresponding to road and pedestrian conditions. The results show that increasing GCI content reduces mass by approximately 9.6 g per 1% GCI, reaching a reduction of about 20% at 50% GCI. For polished surfaces, SRV increased up to 77 (MS55) and 75 (MS96) at 40–45% GCI. For ground surfaces, optimal performance was observed at 10% GCI, while higher contents reduced SRV and caused mechanical degradation above 30–35% GCI. The results indicate that optimized GCI content can improve slip resistance while reducing material weight.

## 1. Introduction

Research on the modification of concrete mixtures through the incorporation of waste additives represents an important direction in the development of construction materials [1]. Among the studied materials containing waste components, two main groups can be distinguished, namely mineral waste [2,3] and plastic waste [4,5,6]. Mineral waste generally exhibits better compatibility with the cement matrix. This results from the similar physicochemical characteristics of the constituents. The literature reports analyses of various types of mineral additives, including industrial sludge [7], ash and slag [8], waste dusts [9], as well as glass additives and silica-based materials [10]. In many cases, these additives enabled the preservation or improvement of selected mechanical properties of concrete.

The application of fillers derived from electrical cable insulation waste in concrete paving blocks constitutes a solution that integrates environmental [11,12], technological [13], and economic objectives [14,15]. These wastes, typically in the form of shredded polymers such as Polyethylene Terephthalate (PET), Polycarbonate (PC), Polyvinyl Chloride (PVC), High-Density Polyethylene (HDPE), and High-Impact Polystyrene (HIPS) [16,17,18,19,20,21], belong to a group of materials that are difficult to process using conventional recycling methods. This is due to their complex composition, the presence of modifying additives, and various contaminants [22]. The incorporation of such materials into a cement-based composite enables their safe and durable utilization. This approach reduces the amount of waste directed to landfills or incineration. It is therefore consistent with the principles of the circular economy.

From the perspective of construction materials engineering, the use of such fillers leads to a significant modification of the physical and mechanical properties of concrete [23,24]. Due to the substantially lower density of plastics compared to mineral aggregates [23,24], it is possible to obtain elements with reduced mass [25]. This is relevant both during transport and during the installation of pavement systems. At the same time, the presence of polymer particles affects the mechanical response of the material. It reduces brittleness and may improve resistance to the initiation and propagation of micro cracks, especially under dynamic loading conditions [26]. This effect results from the partial crack bridging provided by more deformable polymer inclusions [27]. Concrete modified with plastic additives exhibits improved impact resistance and enhanced energy absorption capacity, which makes it more resistant to dynamic loading conditions [23,28]. Concrete containing plastic fillers also shows improved insulating properties, including sound attenuation and thermal insulation, which may be advantageous in construction applications [28]. However, it should be emphasized that the incorporation of plastic waste is associated with certain limitations. The key issue is the weak adhesion between the cement matrix and polymer particles, resulting from chemical incompatibility [29,30], unfavorable surface properties [30,31], and differences in porosity and microstructure [32,33]. This leads to a reduction in compressive strength and elastic modulus of the composite [24,34]. Additionally, an increase in porosity may occur, which can adversely affect the durability of the material [24,35]. For this reason, the content of such fillers should be strictly controlled. Their particle size distribution and potential surface treatment should be optimized to improve interfacial bonding.

Ongoing research focuses on improving concrete structures in which non-conventional fillers derived from electrical cable recycling processes are used [36,37]. However, there is a lack of studies addressing the influence of these fillers on slip risk for pedestrian traffic, as well as for individuals using wheelchairs or mobility aids such as crutches [38].

Slip risk is investigated for both indoor [39,40,41] and outdoor pedestrian surfaces [42,43,44], as well as for roads intended for vehicular traffic [45,46]. The main research focuses on the influence of contaminants on slip resistance, such as with soil [47], water [48] and oil [49,50], the type of surface finishing [51], including treatments applied to stone [52] and wood [39] surfaces, and the effect of protective coatings and impregnating agents [53]. In contrast, concrete surfaces have been studied to a more limited extent [54,55,56], and the available research remains insufficient, particularly in the context of modified concrete incorporating waste-based fillers [57].

The aim of this study is to determine the effect of the volumetric content of fillers derived from electrical cable recycling waste, in the form of plastic insulation, used as a partial replacement for sand (fine aggregate), on the slip resistance of concrete paving blocks.

## 2. Materials and Methods

### 2.1. Measurement Setup

**Figure 1:**
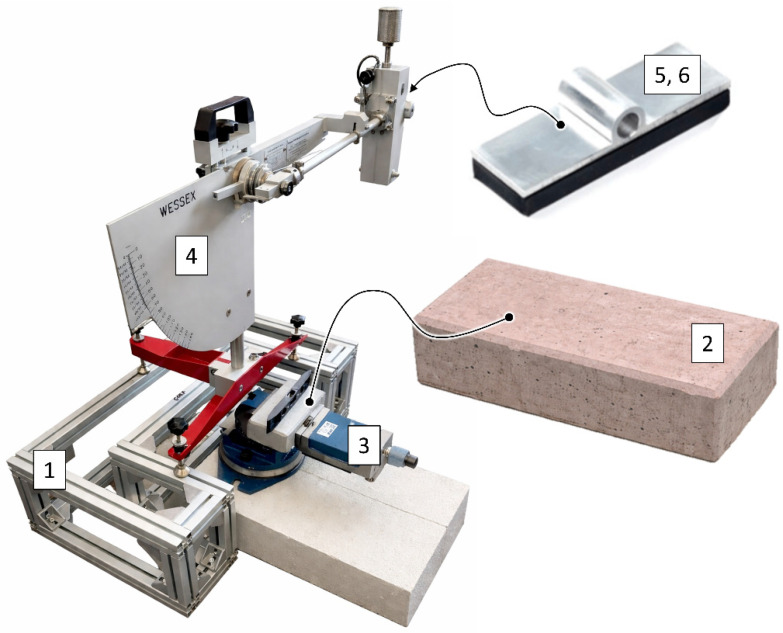
Test rig used in the study, where 1—denotes the rigid frame, 2—the test specimens, 3—the vice, 4—the British Pendulum Tester, 5, 6—the Mounted Shoe 55 and Mounted Shoe 96 sliders, respectively.

The experiments were conducted using a custom designed test rig (Figure 1), consisting of a rigid frame (1) that served as the load bearing structure for the measurement system. Test specimens (2) were mounted in a vice (3), which enabled precise leveling of the surface relative to the pendulum motion axis. The main component of the setup was a British Pendulum Tester (4), model Friction Pendulum Slip Resistance RED TITANIUM (WESSEXlab, Madrid, Spain). The tests were carried out in accordance with AS 4586:2013 Slip Resistance Classification of New Pedestrian Surface Materials, using two types of rubber sliders, namely Mounted Shoe 55 and Mounted Shoe 96. The Mounted Shoe 55 slider (5), with a hardness of 55 ± 4 IRHD according to ISO 48-2:2018, was used for testing surfaces with higher roughness and under wet conditions. This corresponds to its typical application in the assessment of road surface slip resistance. The Mounted Shoe 96 slider (6), characterized by a hardness of 96 ± 2 IRHD, was used for testing pedestrian surfaces, particularly under dry conditions and in outdoor environments. It should be noted that although the Mounted Shoe 55 slider is commonly associated with road surface testing, the British Pendulum Tester does not replicate real tire–road interaction conditions. Therefore, the results obtained in this study should be interpreted as a comparative assessment of surface friction rather than a direct evaluation of performance under actual vehicular loading.

It should be noted that the two sliders represent standardized testing conditions rather than actual contact scenarios. The MS55 slider, due to its lower hardness, is commonly associated with conditions relevant to road surface testing and wet environments, whereas the MS96 slider is more representative of pedestrian-type contact conditions. However, neither slider reproduces real interactions such as tire–road or footwear–surface contact, and therefore the results should be interpreted as comparative rather than directly representative of specific applications. All measurements in this study were performed under wet conditions, which should be taken into account when interpreting the results in relation to real operating conditions.

### 2.2. Tested Specimens

**Figure 2:**
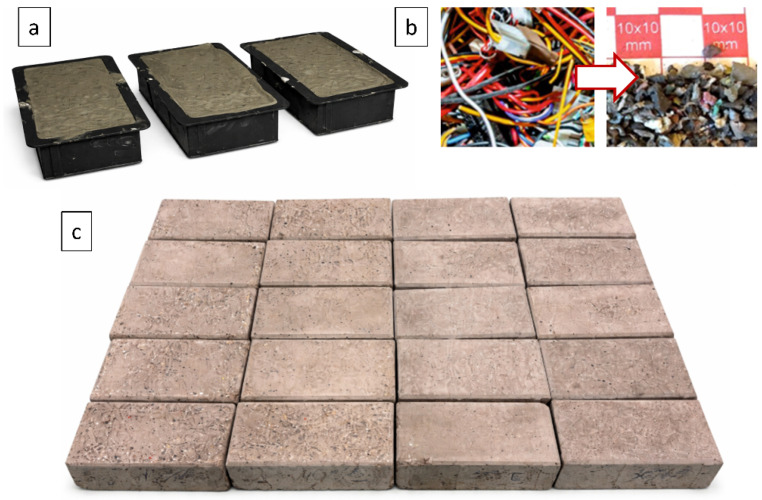
Illustration of materials and paving blocks at different stages of preparation, where (**a**) specimens in molds immediately after mixing, (**b**) insulation material used as a replacement for sand in the concrete mixture, and (**c**) finished paving blocks during curing.

The specimens were produced using standardized molds (Figure 2a), corresponding to rectangular paving blocks in accordance with PN-EN 1338. The obtained elements had dimensions of 200 × 100 × 60 mm and were made of C20/25 class concrete. The mixture proportions were defined on a volumetric basis and consisted approximately of 50% sand (fine aggregate, 0–4 mm), 30% coarse aggregate (0–8 mm mixed aggregate), 16% cement, and 4% water. The corresponding water-to-cement ratio was approximately w/c ≈ 0.5. The concrete was prepared using commercially available Portland cement and natural aggregates. Due to the industrial origin of the specimens, detailed information on cement type, aggregate grading curves, and exact batch quantities was not available. No chemical admixtures were intentionally introduced. The workability of the fresh mixture was adjusted to allow proper casting into molds and was assessed qualitatively as typical for paving block production. This mixture served as the reference composition and was modified by partially replacing the volumetric fraction of sand (fine aggregate) with an equivalent volume of shredded electrical cable insulation. It should be noted that the primary objective of the study was a comparative evaluation of slip resistance as a function of GCI content, rather than the development of an optimized concrete mix design. Therefore, the mixture composition was kept consistent across all specimens, and the influence of GCI content was analyzed relative to the reference material.

The insulation material used (Figure 2b) originated from an industrial recycling process of electrical cables, primarily from automotive installations. It consisted of a heterogeneous mixture of polymers, including PET, PC, PVC, HDPE and HIPS. Due to the mixing of recyclates derived from different cable types, precise determination of the material composition in terms of quantitative polymer fractions was not possible. The material should therefore be treated as a heterogeneous industrial recyclate consisting of an undefined mixture of polymers, including PET, PC, PVC, HDPE, and HIPS. This heterogeneity reflects real recycling conditions but introduces variability in material properties, which may affect the reproducibility and interpretation of the results. This approach reflects typical industrial recycling streams, where material composition is not strictly controlled. The insulation granulate was obtained through a shredding process using a sieve with a mesh size of 1 mm.

Particle size distribution analysis was performed using a mechanical sieve shaker. The dominant fraction ranged from 1 mm to 0.71 mm and accounted for 34.1%. The fraction from 0.71 mm to 0.5 mm accounted for 28.2%, and the fraction from 0.5 mm to 0.25 mm accounted for 28.8%. The extreme fractions above 1 mm and below 0.25 mm accounted for 1.4% and 7.5%, respectively. A detailed analysis of the particle size distribution of the cable insulation material is presented in Marciniak et al. (2024) [22]. The same material was used in the investigated concrete mixtures [22]. However, due to the heterogeneous composition and industrial origin of the recyclate, the bulk density of the GCI material was not directly measured in this study.

A series of paving block specimens (Figure 2c) was prepared with volumetric fractions of shredded insulation equal to 0%, 5%, 10%, 15%, 20%, 25%, 30%, 35%, 40%, 45%, and 50%. For each volumetric level, five identical specimens were produced. An additional set of specimens was prepared and subjected to surface treatment consisting of roughening the top layer by grinding with abrasive paper of grit size 60. However, slip resistance testing was performed only for specimens up to 45% GCI for polished surfaces and up to 35% GCI for ground surfaces, because higher GCI contents led to progressive damage during testing and limited further tribological evaluation.

After casting and demolding, all specimens were cured under laboratory conditions at a temperature of 22 ± 2 °C and a relative humidity of 50% to 60% for a period of 28 days.

The experimental matrix, including the range of GCI contents, number of specimens, and the scope of testing performed for each case, is summarized in Table 1.

It should be noted that although specimens with up to 50% GCI were produced and evaluated in terms of mass, slip resistance testing was limited at higher GCI levels due to progressive damage observed during testing with the British Pendulum Tester.

### 2.3. Experimental Procedure

Prior to testing, each specimen, after completion of the curing process, was weighed and visually inspected to exclude material defects such as cracks and surface inhomogeneities. The test procedure began with mounting the specimen in the vice and leveling its surface. Leveling was performed using a bubble level to ensure proper alignment with the pendulum motion axis.

The pendulum was then positioned in the measurement configuration, and the specimen surface was wetted with water. Measurements were carried out along the longer edge of the paving block, in accordance with BS EN 13036-4:2011, allowing for the determination of the British Pendulum Number (BPN).

During testing, the BPN value was recorded directly from the device. The results were then processed statistically and converted into the Skid Resistance Value (SRV). These values were compared with a four-level qualitative classification scale recommended by the UK Slip Resistance Group, consistent with HB 198:2014 Guide to the Specification and Testing of Slip Resistance of Pedestrian Surfaces (Table 2).

For each specimen, ten BPN measurements were performed using repeated pendulum swings. The extreme values, namely the maximum and minimum, were discarded to reduce the influence of outliers. The arithmetic mean of the remaining eight values was calculated and treated as the representative SRV for a single specimen. The experimental unit in this study was an individual concrete specimen. For each GCI level, five independent specimens were tested (n = 5). The reported mean SRVs were calculated as the average of these specimens. Confidence intervals (*p* = 0.05) were determined based on the variability between specimens, not on repeated pendulum measurements. Thus, the statistical analysis reflects specimen-to-specimen variability rather than measurement repeatability. The repeated pendulum measurements performed on each specimen were used only to obtain a stable representative value for that specimen and were not treated as independent statistical observations. This approach is consistent with standard practice in slip resistance testing, where repeated swings are used to stabilize the measurement, while statistical inference is based on independent specimens.

The obtained results for different volumetric fractions of cable insulation were subjected to statistical analysis using the Student’s t distribution. Based on this analysis, confidence intervals were determined for each SRV value at a significance level of *p* = 0.05.

**Figure 3:**
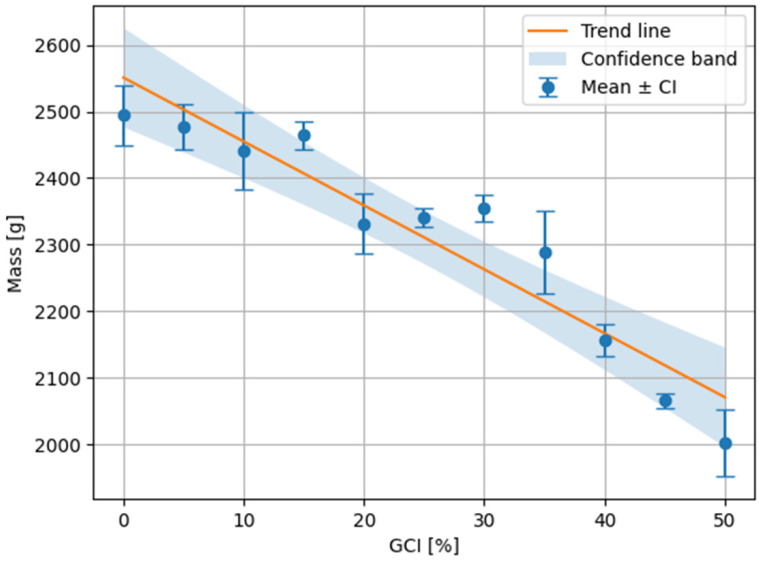
Relationship between the mean mass of concrete paving blocks and the volumetric content of GCI. Error bars represent 95% confidence intervals. The solid line indicates a linear regression used to describe the overall mass trend, and the shaded area denotes the confidence band.

**Figure 4:**
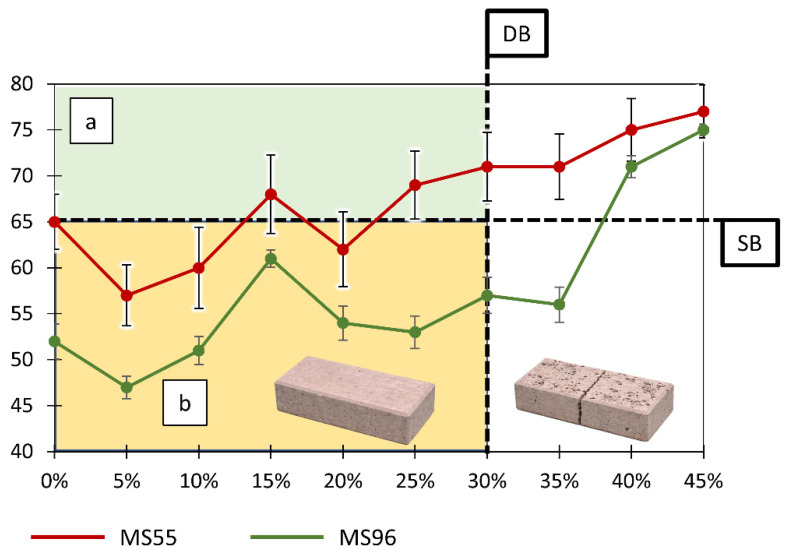
Relationship between slip resistance (SRV) and GCI content for concrete paving blocks with a polished surface. Results were obtained using the British Pendulum Tester equipped with Mounted Shoe 55 and Mounted Shoe 96. The vertical dashed line (DB) indicates a preliminary damage boundary identified based on specimen behavior during testing. It separates specimens that remained intact from those exhibiting visible mechanical degradation caused by repeated interaction with the British Pendulum Tester. This boundary reflects a practical testing limitation rather than a definitive mechanical strength threshold. The horizontal dashed line (SB) corresponds to the slip boundary (SRV ≈ 65), dividing low and extremely low slip risk regions according to the adopted classification scale, used here for comparative purposes only. Region (a) indicates low slip risk (SRV ≥ 65), while region (b) indicates moderate slip risk (SRV < 65).

**Figure 5:**
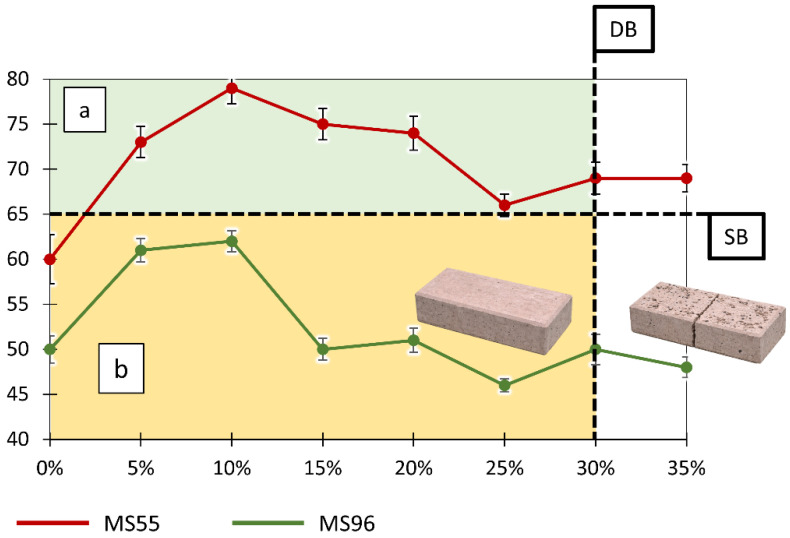
Relationship between slip resistance (SRV) and GCI content for ground (abraded) concrete paving blocks. Results were obtained using the British Pendulum Tester equipped with Mounted Shoe 55 and Mounted Shoe 96. The vertical dashed line (DB) indicates a preliminary damage boundary, beyond which specimens exhibited cracking and material degradation induced during repeated pendulum testing. This boundary denotes the limit of suitability for further slip resistance measurements rather than an intrinsic mechanical failure threshold. The horizontal dashed line (SB) represents the slip boundary (SRV ≈ 65), distinguishing low and extremely low slip risk regions according to Table 2. Region (a) indicates low slip risk (SRV ≥ 65), while region (b) indicates moderate slip risk (SRV < 65).

The confidence intervals (CIs) were calculated using Student’s t-distribution according to (1):

CI = t_0.05_·(s/√n) (1)

where s is the standard deviation of the specimen means and n = 5 is the number of independent specimens. The statistical unit in this analysis was the mean SRV obtained for each individual specimen. Thus, each data point presented in Figures 3–5 corresponds to the mean value calculated from five independent specimens (n = 5).

## 3. Results

The first analyzed parameter was the mass variation in paving blocks as a function of the volumetric content of granulated cable insulation (GCI). The introduction of shredded cable insulation as a partial replacement for sand significantly affects the bulk density of the concrete mixture and, consequently, the mass of the final elements (Table 3, Figure 3).

This effect results from differences in the densities of the constituent materials. The typical density of PVC used in cable insulation ranges from approximately 1300 to 1450 kg/m^3^. PET exhibits a density of 1340 to 1400 kg/m^3^, PC 1180 to 1220 kg/m^3^, HDPE 940 to 970 kg/m^3^, and HIPS 1030 to 1060 kg/m^3^. In contrast, the density of quartz sand is significantly higher, typically in the range of 2600 to 2650 kg/m^3^.

The mean mass of the paving blocks shows an overall decreasing trend with increasing volumetric content of GCI. For the reference sample (0% GCI), the mean mass was 2494 g, whereas for the highest analyzed content of 50% GCI it decreased to 2002 g. Although local deviations from monotonic behavior are observed between some adjacent GCI levels, the general tendency remains downward. A linear regression model (2) was used only as a simplified approximation of the overall trend, indicating an average mass reduction of approximately 9.6 g per 1% increase in GCI content over the analyzed range. This corresponds to an overall decrease of approximately 492 g between 0% and 50% GCI.

(2) m=−9.59⋅GCI+2508.7

For the individual measurement series, the mean values fall within the confidence intervals determined for a significance level of *p* = 0.05. The width of these intervals ranges from approximately ±10.8 g to ±62.1 g, depending on the volumetric content of GCI. Despite local deviations in the mean values, particularly in the range of 10% to 30% GCI, the overall tendency is decreasing, although the relationship is not strictly monotonic.

Additionally, it was observed that specimens with a GCI content of 35% represent a threshold level at which the elements did not undergo damage during testing with the British Pendulum Tester. For specimens with higher GCI content, the interaction with the pendulum, in the form of sliding contact, led to material damage such as cracking or fragmentation.

In the next stage, the SRV was evaluated for two surface conditions, namely a new polished surface (Table 4) and a ground surface (Table 5). The analysis of the results indicates a significant influence of both the GCI content and the surface condition on the slip resistance properties of the tested paving blocks.

For the polished surface (Table 4), formed as a result of the replication of the smooth mold surface by the cement paste settling at the bottom of the mold during casting, a general increasing trend of SRVs with increasing GCI content is observed. Fluctuations in SRVs within the range of 0–10% GCI (e.g., a decrease from 65 to 57 and 60 for MS55, and from 52 to 47 and 51 for MS96) fall within the standard deviation and should be interpreted as statistical variability rather than a change in trend.

For GCI contents above 10%, a systematic increase in SRV is observed, leading to values corresponding to the “extremely low slip risk” category according to the adopted classification (≥65), particularly for contents of 40–45% (77 for MS55 and 75 for MS96). It should be noted that this classification provides a comparative description of slip resistance under controlled laboratory conditions and does not by itself establish suitability for practical applications. This effect can be tentatively attributed to the progressive disruption of the continuity of the smooth cement paste layer. At low GCI contents, the surface retains the characteristics of a uniform and smooth cement film, which under wet conditions promotes the formation of a continuous water layer and reduces friction. With increasing GCI content, the continuity of this layer is gradually lost, leading to the development of micro-roughness and the disruption of the water film, which may in turn contribute to an increase in SRV.

Considering both the SRV results (Table 4) and observations related to the mechanical durability of the specimens, it is possible to define a material resistance boundary denoted as DB (damage boundary), as shown in Figure 4. This boundary, represented as a vertical line, divides the GCI content into two regions. On the left side of the DB, corresponding to contents up to approximately 30–35% GCI, the specimens maintain structural integrity and remain suitable for slip resistance testing without visible damage. On the right side, higher GCI contents lead to material degradation, manifested by cracking and fragmentation.

A second important interpretative boundary is the horizontal SRV limit denoted as SB (slip boundary), also shown in Figure 4, corresponding to SRV ≈ 65 according to Table 2. This line divides the plot into regions of low and extremely low slip risk.

It should also be noted that the results for the polished surface (Table 4, Figure 4) exhibit greater variability, as reflected by wider confidence intervals, for example ±3 to 4 for MS55. This indicates lower stability of the slip resistance properties of the near-surface layer formed by the cement paste, which is highly sensitive to local microstructural variations and the presence of a water film.

Figure 5 presents the relationship between SRV and GCI content for ground (abraded) concrete paving blocks.

For the ground surface (Table 5), a different trend is observed. In the range of low GCI content, up to 10%, a significant increase in SRV is recorded, from 60 to 79 for MS55 and from 50 to 62 for MS96. This corresponds to a transition into the extremely low slip risk range (Table 2). This effect may result from the introduction of additional micro-roughness by GCI particles exposed after grinding with P60 abrasive paper.

At higher GCI contents, greater than or equal to 15%, a decrease in SRV is observed, reaching 66 to 69 for MS55 and 46 to 50 for MS96. This corresponds to a return to the low slip risk range. This behavior is associated with the increasing contribution of the polymer phase, which has lower hardness and leads to a reduction in effective micro-roughness.

Similarly to the polished surface, a damage boundary DB can be identified (Figure 5), separating specimens that maintain structural integrity from those that undergo mechanical degradation, which prevents further grinding. The slip boundary SB, corresponding to SRV ≈ 65, separates the slip risk ranges.

Despite the use of two different sliders, Mounted Shoe 55 and Mounted Shoe 96, the relationships between SRV and GCI content show a very similar trend for both surface conditions (Table 4 and Table 5, Figure 4 and Figure 5). Geometric analysis of the curves indicates that the SRV versus GCI relationships have analogous shapes for both sliders, with the MS55 curve shifted upward relative to MS96. For the polished surface, the absolute difference ranges from 2 to 16 SRV units, most commonly between 7 and 15 SRV. For the ground surface, the difference ranges from approximately 10 to 25 SRV units.

Despite the use of two different sliders, Mounted Shoe 55 and Mounted Shoe 96, the relationships between SRV and GCI content show a very similar trend for both surface conditions. This indicates that the observed changes are primarily governed by surface structure. The differences in absolute SRVs between the sliders are associated with their hardness and contact characteristics. However, these differences should be interpreted within the framework of standardized testing conditions and do not directly correspond to specific real-world contact situations.

Despite this difference in absolute values, the characteristic points of the curves, including increases and decreases in SRV, occur at the same ranges of GCI content. This indicates that the shape of the SRV versus GCI relationship is primarily governed by the surface structure, while the type of slider affects only the absolute level of SRV without altering the overall trend.

The observed trends are consistent across both sliders; however, their interpretation in the context of vehicular applications remains limited due to the simplified contact conditions inherent to the BPT method.

## 4. Discussion

The incorporation of plastic waste into concrete as a partial replacement for conventional aggregates, either fine or coarse, leads to a reduction in the overall mass of the material. This reduction results primarily from the lower density of plastic materials compared to natural aggregates such as sand or gravel. The replacement of a portion of sand with GCI in the mixture used for paving block production results in a significant decrease in the mass of the final product, amounting to approximately 9.6 g per 1% increase in the volumetric content of GCI. The non-monotonic differences observed between some adjacent GCI levels are most likely associated with the natural variability of concrete-based composites and the heterogeneous character of the recycled insulation filler. This finding is consistent with the results reported by Mahzuz and Tahsin (2019) [58], who demonstrated that replacing natural aggregates with plastic waste reduces the density of concrete. For example, substituting 25% and 50% of coarse aggregate with HDPE resulted in a reduction in concrete unit weight by 9.8% and 12.4%, respectively. This effect is attributed to the lower density of plastic materials compared to traditional mineral aggregates.

At the same time, the addition of GCI weakens the bonding within the material structure after the concrete curing process, which leads to a deterioration of its mechanical properties. When the volumetric content of GCI exceeds approximately 35%, the produced paving blocks no longer meet functional requirements due to excessive fragmentation and increased susceptibility to cracking. This observation is consistent with findings reported in the literature on concrete modified with plastic waste. Odaa et al. in 2023 [59] and El-Nadoury in 2022 [60] demonstrated that the incorporation of plastic, particularly in the form of particles or fibers, weakens the interfacial bonding between the cement matrix and the polymer phase. This is mainly due to the smooth surface of plastic materials, which reduces adhesion and leads to a decline in mechanical performance [59,60]. El-Nadoury in 2022 further showed that chemical modification of plastics, such as increasing surface roughness and hydrophilicity, can improve interfacial bonding [60]. Moreover, El-Nadoury in 2022, Wu et al. in 2020, and Devi in 2024 [35,60,61] reported that increasing plastic content in concrete leads to a reduction in compressive and tensile strength, particularly at higher plastic fractions. The optimal plastic content is typically around 5%, which may improve selected mechanical properties, while higher proportions result in structural weakening. It has also been shown that the addition of plastic up to approximately 5% by cement mass can enhance compressive and tensile strength, whereas exceeding this threshold slows strength development and increases cracking susceptibility. It should be noted that results reported for plastic fibers, such as polypropylene fibers, are not directly comparable to the present study, as fiber-reinforced systems involve different reinforcement mechanisms (e.g., crack bridging) compared to granular plastic fillers used as aggregate replacement [62,63].

From the perspective of fracture mechanics, the observed degradation at higher GCI contents can be associated with several interacting mechanisms. The introduction of polymer particles into the cementitious matrix creates a heterogeneous structure with weak interfacial transition zones between the polymer and the cement paste. Due to the low stiffness and poor adhesion of the polymer phase, these interfaces act as preferential sites for crack initiation. Under repeated local loading conditions, such as those imposed by the British Pendulum Tester, microcracks may initiate at these interfaces and propagate through the surrounding matrix. With increasing GCI content, the distance between inclusions decreases, facilitating microcrack coalescence and leading to the formation of continuous crack paths. This results in a transition from a dispersed microcracking regime to a more pronounced damage state characterized by fragmentation and loss of integrity. At lower GCI contents, the presence of polymer particles may contribute to increased surface roughness and improved tribological performance. However, beyond a critical threshold, this beneficial effect is outweighed by the formation of structural discontinuities, which reduce the load-bearing capacity of the material and promote damage under repeated contact. A more rigorous assessment of these mechanisms would require dedicated experimental and numerical approaches, including fracture-oriented mechanical testing (e.g., fracture energy, crack propagation resistance) and modelling techniques capable of capturing crack initiation and evolution in heterogeneous composites. Such approaches have been successfully applied in the literature to analyze fracture processes in complex materials and could provide a deeper understanding of the damage boundary identified in this study [64,65].

In the case of specimens with a polished surface, the presence of a cement paste layer, referred to as laitance, resulted in a deterioration of slip resistance properties. This smooth layer, replicating the surface of the mold, exhibited poorer tribological performance compared to surfaces containing exposed insulation particles. Consequently, the addition of GCI may lead to an improvement in slip resistance by disrupting the continuity of this layer. Laitance is a weak, powdery layer that forms on the surface of fresh concrete due to the segregation of fine particles and water rising to the surface during the setting process [66]. This phenomenon is influenced by factors such as mix design, water content, and placement conditions. The observations obtained in this study are consistent with the findings of Kim in 2022 [54], who confirmed that the presence of laitance can alter the surface texture of concrete and reduce its slip resistance. Smooth or powdery laitance layers often lack sufficient friction, particularly under wet or contaminated conditions. Slip resistance is strongly dependent on surface finish. Rougher textures generally provide improved traction, although this relationship is not always linear, as factors such as contamination, including water, oil, or soapy substances, may significantly reduce friction even for rough surfaces. Liu et al. in 2021 [67] indicated that controlling the rheological properties of fresh concrete and ensuring proper compaction can reduce segregation and limit the formation of laitance.

It should be emphasized that the proposed explanations of the observed tribological behavior, including disruption of the cement paste layer (laitance), exposure of GCI particles, and changes in surface micro-roughness, are based on indirect interpretation of SRV results and literature data. No direct surface characterization methods, such as profilometry, microscopy, or quantitative roughness measurements, were applied in this study. Therefore, these mechanisms should be treated as plausible hypotheses rather than confirmed explanations. Further research involving detailed surface characterization is required to validate these interpretations.

For ground surfaces, the beneficial effect of increased roughness is observed up to a GCI content of approximately 10%. Beyond this level, a gradual loss of favorable properties related to the interaction between the concrete matrix and insulation particles is observed. The tribological contact becomes increasingly governed by GCI particles, which are characterized by lower hardness and greater deformability compared to mineral aggregates. As their content increases, the surface undergoes effective smoothing, and its behavior becomes similar to that of an unpolished surface covered with a layer exhibiting properties comparable to cement laitance, resulting in a decrease in SRV. Chen and Hong in 2016 and Subedi et al. in 2025 [68,69] reported similar relationships, indicating that surface grinding improves the texture of concrete by enhancing macrotexture and increasing slip resistance. Grinding also leads to an immediate improvement in frictional performance, which reduces the risk of hydroplaning under wet conditions. However, although grinding enhances slip resistance, these effects may degrade over time due to traffic loading and surface wear.

The use of two types of sliders, Mounted Shoe 55 and Mounted Shoe 96, enabled a comprehensive evaluation of the slip resistance properties of the material. The obtained SRVs indicate that the material may be suitable for application in pedestrian surfaces and, within a defined range, also in road surfaces.

The use of two sliders with different hardness values provides a broader comparative assessment of frictional behavior; however, their interpretation in terms of real applications should be approached with caution. The MS55 slider is typically associated with conditions relevant to road surface evaluation, particularly under wet conditions, while the MS96 slider is more representative of pedestrian-type contact. Nevertheless, both sliders operate under simplified and controlled conditions and do not replicate key aspects of real interactions, such as tire deformation, footwear material variability, contact pressure distribution, or dynamic effects. In addition, all tests in this study were conducted under wet conditions, which further limits direct extrapolation to dry or mixed service environments.

The identified damage boundary (DB), located at approximately 30–35% GCI, should be interpreted as a testing-related threshold rather than a practical application limit. It reflects the point at which specimens were no longer able to withstand repeated interaction with the British Pendulum Tester without undergoing visible damage, which prevented further slip resistance measurements. The observed damage, including cracking and surface fragmentation, resulted from repeated dynamic loading imposed by the pendulum slider.

This boundary does not represent a structural or operational limit of the material. In practical applications, the acceptable content of plastic fillers in concrete is typically much lower, often in the range of 5–10%, depending on the required mechanical performance and durability. Therefore, the higher GCI levels investigated in this study should be understood as part of an extended experimental range, used to identify trends and limits of behavior, rather than as realistic application scenarios. Additional standardized mechanical tests, such as compressive strength, abrasion resistance, and fatigue performance, are required to define reliable structural limits. However, further mechanical testing is required to clearly define the maximum allowable GCI content that ensures compliance with relevant standards, particularly with respect to load resistance under wheel loading conditions. According to findings reported in the literature, the practical DB limit for industrial applications is likely to be significantly lower, typically around 5–10% of plastic filler content [70,71].

The SRV classification used in this study provides a standardized framework for describing slip resistance levels; however, it should not be interpreted as a direct indicator of application suitability. The classification is based on controlled laboratory conditions and does not account for important factors influencing real-world performance, such as surface wear, contamination (e.g., water, oil, or dust), aging, and environmental exposure. Therefore, the results should be treated as indicative of relative performance rather than as a definitive assessment of safety under service conditions.

An important limitation of the present study is the heterogeneous nature of the recycled cable insulation used as a filler. The material consists of a mixture of different polymers with varying mechanical properties, surface energies, and hardness, and its exact composition is not quantitatively defined. This may lead to local variability in the polymer–cement interface and in the surface microstructure exposed during grinding. Consequently, the observed tribological behavior should be interpreted as representative of a mixed recyclate rather than a single, well-defined material system. This limitation may also influence the repeatability of results and should be considered when comparing with studies based on homogeneous polymer additives.

In general, the incorporation of recycled electrical cable insulation reduces slip risk, which is consistent with findings reported in studies analyzing the influence of plastic fiber additives [72] and plastic waste materials [73]. A similar behavior was also observed in the present study, where excessive plastic content in the concrete mixture led to the formation of voids within the structure. This phenomenon may reduce the overall quality and durability of the material [72].

## 5. Conclusions

The present study investigated the effect of the volumetric content of recycled electrical cable insulation used as a partial replacement of fine aggregate on the slip resistance of concrete paving blocks. The research question addressed whether the incorporation of such waste materials influences surface safety properties, primarily for pedestrian applications, while the potential applicability to vehicular conditions remains preliminary and requires further validation using tire-based testing methods. The results indicate that increasing the content of GCI leads to a systematic reduction in the mass of concrete elements, with an average decrease of approximately 9.6 g per 1% increase in GCI content. In terms of tribological performance, the effect of GCI strongly depends on the surface condition. For polished surfaces, an increase in GCI content results in an increase in slip resistance under laboratory conditions, due to the disruption of the continuous cement paste layer. This improvement is reflected in the SRVs and their classification; however, this classification should be interpreted as descriptive and not as a direct measure of application suitability, which requires further investigation under realistic service conditions. For ground surfaces, the optimal improvement is observed at low GCI contents, up to approximately 10%, beyond which slip resistance decreases due to the increasing dominance of the polymer phase. Additionally, a damage boundary was identified at approximately 30–35% GCI, above which the material exhibits significant mechanical degradation (preventing testing with the British Pendulum Tester).

The study contributes to the existing literature by providing a combined analysis of material composition and surface tribological behavior, which has not been previously addressed for recycled cable insulation in concrete. In particular, it introduces the concept of combined evaluation of slip resistance and observed material degradation during testing, including the definition of slip and damage boundaries. This approach is relevant for the design of safe and sustainable pavement materials, especially in the context of circular economy strategies. However, the study has several limitations. The mechanical properties of the material were assessed primarily through qualitative observations rather than standardized strength tests. In addition, the slip resistance analysis was limited to laboratory conditions and did not account for long-term operational effects such as wear, contamination, or environmental degradation. It should be noted that the results were obtained for a heterogeneous mixture of recycled polymers, and the variability of this material may influence both tribological behavior and repeatability. Future research should focus on comprehensive mechanical characterization of the material, including compressive strength, abrasion resistance, and fatigue behavior under realistic loading conditions. It is also recommended to investigate long-term durability and surface degradation in real operational environments. Further studies should explore optimization of particle size distribution and surface modification of GCI to improve interfacial bonding and expand the safe application range of such materials. The study does not address structural load-bearing performance; therefore, the suitability of the material for road or wheel-loading applications requires separate mechanical validation. The interpretation of surface-related mechanisms is based on indirect evidence and requires confirmation through dedicated surface characterization methods.

## Figures and Tables

**Table 1:** Experimental matrix of specimens and test scope.

GCI Content [%]	Number of Specimens (n)	Mass Measurement	Slip Resistance—Polished Surface	Slip Resistance—Ground Surface
0	5	Yes	Yes	Yes
5	5	Yes	Yes	Yes
10	5	Yes	Yes	Yes
15	5	Yes	Yes	Yes
20	5	Yes	Yes	Yes
25	5	Yes	Yes	Yes
30	5	Yes	Yes	Yes
35	5	Yes	Yes	Yes
40	5	Yes	Yes	No (damage during testing)
45	5	Yes	Yes	No (damage during testing)
50	5	Yes	No	No

**Table 2:** Qualitative slip risk classification based on SRVs.

Slip Risk	SRV
High	≤25
Moderate	25–35
Low	35–65
Extremely low	≥65

**Table 3:** Mean mass values of paving blocks as a function of GCI content, expressed as mean ± confidence interval (*p* = 0.05), based on n = 5 specimens.

GCI, %	Mean Mass, g (*p* = 0.05)
0	2494.2 ± 45
5	2477.0 ± 34
10	2441.4 ± 58
15	2464.2 ± 21
20	2330.8 ± 45
25	2340.6 ± 15
30	2355.4 ± 20
35	2289.2 ± 62
40	2156.6 ± 24
45	2065.6 ± 11
50	2002.0 ± 50

**Table 4:** SRV of polished concrete paving blocks as a function of GCI content.

GCI [%]	Mounted Shoe 55 SRV (*p* = 0.05)	Mounted Shoe 96 SRV (*p* = 0.05)
0	65 ± 3	52 ± 2
5	57 ± 3	47 ± 2
10	60 ± 4	51 ± 2
15	68 ± 4	61 ± 2
20	62 ± 3	54 ± 3
25	69 ± 3	53 ± 3
30	71 ± 3	57 ± 3
35	71 ± 3	56 ± 3
40	75 ± 3	71 ± 2
45	77 ± 3	75 ± 2

**Table 5:** Slip resistance (SRV) of ground (abraded) concrete pavers as a function of GCI content.

GCI [%]	Mounted Shoe 55 SRV (*p* = 0.05)	Mounted Shoe 96 SRV (*p* = 0.05)
0	60 ± 3	50 ± 1
5	73 ± 2	61 ± 1
10	79 ± 2	62 ± 1
15	75 ± 2	50 ± 1
20	74 ± 2	51 ± 1
25	66 ± 1	46 ± 1
30	69 ± 2	50 ± 2
35	69 ± 2	48 ± 1

## Data Availability

The original contributions presented in this study are included in the article. Further inquiries can be directed to the corresponding author.
